# The utilisation of tools to facilitate cross-border communication during international food safety events, 1995–2020: a realist synthesis

**DOI:** 10.1186/s12992-021-00715-2

**Published:** 2021-06-24

**Authors:** Carmen Joseph Savelli, Raul Fernando Garcia Acevedo, Jane Simpson, Céu Mateus

**Affiliations:** 1 World Health Organization, Nutrition and Food Safety, Avenue Appia 20, 1211 Geneva, Switzerland; 2 Lancaster University, Faculty of Health and Medicine, Division of Health Research, Bailrigg, Lancaster, LA1 4YW UK

**Keywords:** Realist synthesis, Contextual factors, Food safety, Foodborne disease outbreaks, International networks, Communication tools, Emergency response

## Abstract

Efficient communication and coordination are needed between countries to prevent, detect and respond to international food safety events. While communication tools, networks and systems exist, current evidence suggests that they are only useful within particular contexts and several only target specific geographic areas. There is a need to unpack and explore the mechanisms of how and in what context such communication tools and their components are effective at facilitating international communication and coordination to keep food safe and mitigate the burden of foodborne disease around the world.

A realist synthesis was undertaken to understand how and why certain processes and structures of communication tools, used during international food safety events, influence their utility and effectiveness according to different contextual factors. The focus of this review was explanatory and aimed to develop and refine theory regarding how contextual factors trigger specific processes and mechanisms to produce outcomes. Using the realist context–mechanism–outcome configuration of theory development, a range of sources was used to develop an initial programme theory, including the authors’ experience, a scoping review of published papers and grey literature and input from an expert reference committee. Literature was then systematically located and synthesised from several databases with input from the expert reference committee to refine the programme theory.

The programme theory developed indicates that when a country has interests in food import or export, has the technical infrastructure to detect and respond to food safety events, and is governed in accordance with regional and/or global laws and regulations relating to food control and global health security, then specific mechanisms will facilitate various outcomes. Mechanisms include trust, experience, support, awareness, understanding, a sense of community, standardisation and intersectoral collaboration. The outcomes include using communication tools to relay information abroad and the prevention of foodborne diseases, among others.

Components of such communication tools may be adapted according to different contextual factors to promote, support and improve their use. Improving international coordination and communication during international food safety events is in the interest of global health security and can mitigate the global burden of foodborne disease.

**Supplementary Information:**

The online version contains supplementary material available at 10.1186/s12992-021-00715-2.

## Background

### Rationale for the review

Access to sufficient amounts of safe and nutritious food is an essential requirement for human health. Unfortunately, unsafe food is known to cause more than 200 acute and chronic diseases worldwide, ranging from diarrhoea to cancer [1]. In 2015, the World Health Organization (WHO) reported the first estimates of the global burden of foodborne diseases, indicating that 31 hazards (including bacteria, viruses, parasites, toxins and chemicals) were responsible for 600-million cases of foodborne diseases and 420,000 deaths worldwide in 2010 [2]. Children under five years of age were found to be disproportionately burdened, accounting for 40% of foodborne disease cases, including 125,000 deaths [2]. Foodborne diseases are observed worldwide; however, the African, South-East Asian, and Eastern Mediterranean regions report the highest burden [2]. Unsafe food presents additional consequences in such high-burden areas by impeding socio-economic development, overloading strained or fragile healthcare systems and damaging national economies, trade and tourism [3]. Specifically, a 2018 study by the World Bank indicates that unsafe food costs low- and middle-income economies approximately USD 100 billion in lost productivity and medical expenses each year [4].

Foodborne diseases are preventable; however, prevention requires adequate investment and coordinated action across multiple sectors to strengthen national food control systems. Multiple agencies responsible for food safety, health, agriculture, biosecurity, veterinary services, trade, and several others must work together to build a strong and resilient national food control system [3]. The WHO has identified core capacities that national governments should develop to safeguard national food supplies and contribute to global health security. For example, countries should have the capacity to detect, investigate and respond to food safety events that may constitute a public health emergency of national or international concern promptly through collaboration between the relevant authorities [5]. On an annual basis, the WHO evaluates the development of such core capacities to determine if each country has established functional mechanisms for the detection, prevention and response to foodborne disease and food contamination events. Data from 2018 indicate that 60% of the attributes of core capacities required for food safety have been developed globally, although disparities exist between regions. For example, 74% of the required core capacities have been achieved in Europe, while only 40% of the core capacities have been achieved in Africa [6].

The evolving nature of foodborne disease threats necessitates cross-border coordination to ensure that local foodborne disease outbreaks or food safety incidents can be contained before evolving into an international food safety event. An international food safety event results when unsafe food produced in one country is exported to at least one other country. Even in countries with well-developed capacities related to food safety, past international food safety events have demonstrated that unsafe foods produced abroad and imported for domestic consumption have the potential to result in large-scale outbreaks of foodborne disease. Table 1 illustrates a selection of notably significant and relatively recent food safety events [7–13]. While truly global food safety events happen relatively infrequently, smaller-scale events occur regularly, involving a few countries each time [14, 15]. Such events illustrate that even the most advanced food control systems do not eliminate all foodborne hazards from reaching the public and further highlight the importance of preparedness and having clear governance structures in place to guide international prevention, detection and response activities. The globalisation of our food supply means that unsafe food originating from one country can undoubtedly result in cases of foodborne disease in others.

**Table 1:** Examples of recent large-scale food safety events

Year	Hazard	Food	Geographic scope	Public health impact	Reference
2008	melamine	milk products from China	Products from were directly exported or secondarily distributed to 47 countries worldwide	~ 300,000 infants and children became ill in China, and six died	[7]
2011	*Escherichia coli* O104	fenugreek sprouts from Egypt	Products distributed to Germany and France	~ 4000 people became infected with enterohaemorrhagic *E.coli,* and ~ 800 developed haemolytic uremic syndrome, mainly in Germany but also in France.	[8]
2012	Norovirus	frozen strawberries from China	Products were distributed to Germany	~ 11,000 cases of norovirus infection in Germany were reported, primarily among school children and children in care facilities	[9]
2013/2014	Hepatitis A virus (HAV)	traceback could not indicate a single point source of contamination; blackberries from Bulgaria and redcurrants from Poland identified as the most common ingredients in the lots of berries associated with cases.	Products exported to at least 13 European countries	~ 1500 cases of HAV infection in 13 European countries were identified	[10]
2017/2018	*Listeria monocytogenes*	ready-to-eat meat products (polony) from South Africa	Products were exported to 15 countries in Africa	~ 1000 cases of Listeriosis in South Africa, including 200 deaths.	[11]
2017/2018	*Salmonella* Agona	infant formula from France	Products were exported or secondarily distributed to more than 80 countries worldwide	37 infants infected with salmonellosis in France	[12]
2018	*Listeria monocytogenes*	Frozen vegetable products	Products were exported or secondarily exported to more than 120 countries worldwide	47 cases of Listeriosis across five countries, including nine deaths	[13]

Global food trade grew almost threefold from 2005 to 2015 [16] PP PPand will continue to rise according to projections [17], even in the face of the ongoing global COVID-19 pandemic, during which time the agri-food sector has displayed more resilience to the crisis than other sectors [18]. Thus, there is a need for international coordination to facilitate rapid and efficient communication and collaboration between public health and food safety authorities (i.e. competent authorities) worldwide to prevent, detect and respond to international food safety events when internationally traded food is considered unsafe. Timely mechanisms to facilitate such global communication did not exist until relatively recently. In the early 2000s, WHO Member States recognised this gap and adopted resolutions at the World Health Assemblies in 2000 [19] and 2002 [20] PP PPcalling for improved communication and coordination during international food safety events, including better tools to facilitate this. Since then, advancements in communication technology have facilitated the development or expansion of international networks and knowledge-sharing platforms to exchange molecular subtyping information on foodborne pathogens, epidemiologic information about foodborne diseases, as well as information on food contamination and related traceability details. Throughout this review, the term ‘communication tool’ is used to encompass networks, knowledge-sharing platforms, technical programmes, or systems that facilitate communication related to food safety across national borders. These communication tools are complex for several reasons, including because they represent disparate systems that may or may not interface with each other, operate in different languages, are coordinated by different institutions in different countries and are at various stages of development. Evidence from practice suggests that such tools are only effective within certain contexts and several only target certain geographic areas [14, 21–24]. It is therefore necessary to unpack and explore the mechanisms of how and in what context such communication tools and their components are effective in facilitating international communication and coordination.

Unfortunately, limited research on the attributes and effectiveness of the tools to facilitate cross-border communication during international food safety events has been conducted. As such, existing literature provides limited guidance for decision-makers coordinating international programmes that facilitate information exchange on food safety, on how to adopt best practices to achieve their objectives. Additionally, as explained by Savelli et al. [15], the global food safety community would benefit from examining the interlinkages between such programmes and networks to understand better how they are being used, by whom and in what contexts. Realist synthesis to begin to address this gap is therefore proposed. The main question to guide this research is: how do different tools facilitate cross-border communication during international food safety events, why are they used, by whom, and for what purpose? A realist approach to conduct this review was chosen as it is well suited for the examination of complex programmes through its focus on outcomes in real-world settings and the contextual factors that influence them [25]. This interpretative method is theoretically driven and allows the synthesis of evidence from a range of sources and study designs. The use of theory facilitates a more profound understanding concerning policy intentions and appreciates the complexity of programmes by including the context in the analysis, more so than other review methods [25, 26]. The overall intent of a realist review is the development and refinement of programme theory to understand how context influences mechanisms to generate outcomes. Mechanisms can be understood as the underlying context-dependent processes, behaviours, structures, values or levers that can generate outcomes. The context includes the social, cultural, institutional, historical and environmental factors that form the setting in which actions are taken to trigger mechanisms. The resulting outcomes of the programme, system or intervention under examination are the products of specific mechanisms being triggered in certain contexts [25].

In this review, outcomes are referred to as either first- or second-level outcomes. The first-level outcome of interest is the use of different tools to communicate internationally about issues related to food safety in an efficient manner. The second-level outcomes of interest are the outcomes or consequences of using the tools (e.g. identification of the source of an outbreak, facilitation of risk management actions in different countries, and prevention of foodborne disease). Although important, it is beyond the scope of this review to examine and measure the impact of using different tools on the overall safety of the global food supply.

### Objectives and focus of the review

The primary aim of this synthesis is to address the question: how do different tools facilitate cross-border communication during international food safety events, why are they used, by whom, and for what purpose? The overall objective is to refine a programme theory that explains the contexts (C) in which certain mechanisms (M) generate specific outcomes (O) by developing a C-M-O framework. This programme theory should prove useful to programme coordinators to promote and support the use of communication tools and improve their effectiveness. The specific objectives are as follows:

1. Document the different tools used to facilitate cross-border communication during international food safety events;
2. Identify the contextual factors that trigger mechanisms to influence the outcomes observed in relation to the use of different communication tools;
3. Identify and explain the mechanisms that influence the outcomes observed in relation to the use of different communication tools;
4. Examine the outcomes observed in relation to the use of different communication tools; and
5. Refine a realist programme theory that synthesises review findings and input from an expert reference committee to explain how different tools facilitate cross-border communication during international food safety events, why they are used, by whom, and for what purpose.

## Main text

### Review process

**Fig. 1:**
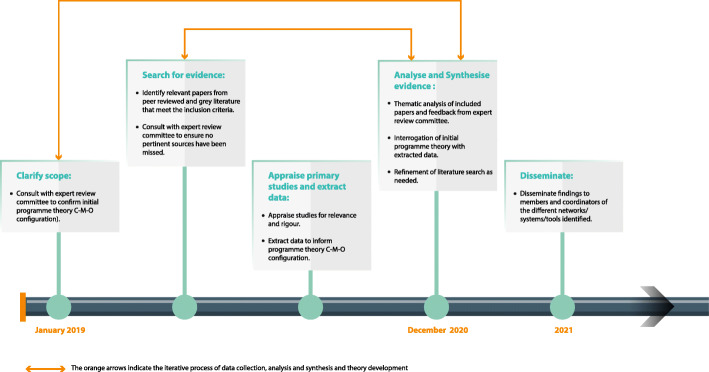
Overview of the stages of this review

This realist synthesis has followed the 2005 protocol provided by Pawson45T45T, Greenhalgh, Harvey, and Walshe45T45T for conducting realist reviews [25] and reporting is guided by the Realist and Meta-narrative Evidence Synthesis: Evolving Standards (RAMESES) from 45T45TWong, Greenhalgh, Westhorp, Buckingham, and Pawson [26]. 45T45T The five steps for conducting a realist review according to Pawson et al. [25] have been followed: 1) clarify scope; 2) search for evidence; 3) appraise primary studies and extract data; 4) analyse and synthesise evidence; and 5) disseminate. While presented sequentially, these steps were iterative and were revisited throughout the review process when new evidence emerged that could contribute to theory refinement (Fig. 1). The grand level development theories that provide an overarching framework for this review include the third wave of modernisation theory developed in the 1990s [27, 28] and globalisation theory as articulated by Robinson [29] and are further described in our published review protocol [30]. With these overarching theories in mind, and using the realist approach, a refined programme theory to explain a context-mechanism-outcome (C-M-O) configuration related to the use of communication tools to facilitate information exchange during international food safety events has been developed. In line with the RAMESES reporting standards, complete information on: 1) the rationale for using realist synthesis; 2) the initial scoping literature search; 3) the searching processes; 4) the selection and appraisal of documents; 5) the data extraction; and 6) the analysis and synthesis processes, are all described in detail in the published review protocol [30] and thus not repeated here. Details on the specific database searches are included in supplementary file 1. There were two changes in the review process described in the published protocol. Firstly, the duration was initially planned as a 12-month period and later revised to a 24-month period due to competing priorities of the first author, CJS. Secondly, selection and appraisal of documents were conducted such that CJS screened the title and abstract of the searched articles using the inclusion and exclusion criteria (as defined in the review protocol [30]). When unsure of acceptability, a second investigator (second author, RFGA) was consulted. If it was unclear from the title and abstract if a paper should be included (or if the paper did not have an abstract as with many documents from grey literature), the full text was reviewed prior to exclusion. Decisions on included and excluded texts were discussed by CJS and RFGA until consensus was reached. The expert reference committee was also engaged in dialogue with the reviewers during selection and appraisal in an effort to include all relevant data from 1995 to July 2020. The eleven expert reference committee members (see acknowledgements) include coordinators of international communication tools as well as individuals who have been involved in their development. The authors made efforts to be inclusive, diverse, and regionally representative when assembling the expert reference committee, whose members’ hail from North America, South America, Europe, the Middle-East and Asia, and many of whom work in international organizations or have extensive international experience. When developing the initial programme theory, CJS was informed by his professional experiences as part of the WHO Secretariat of the International Food Safety Authorities Network (INFOSAN). These experiences have influenced his positionality as an international civil servant valuing international collaboration in the pursuit of a more equitable and healthier world. In particular, facilitating national and regional workshops related to international communication on food safety matters, including in the Americas, Europe, Africa, the Middle-East and Asia, provided a wealth of knowledge from national experts on challenges and lessons learned related to international communication on food safety matters which informed the initial theory development. These activities were carried out with regional counterparts, for example with experts from the WHO Regional Office for Africa and the African Union. While these experts were not formally part of the expert review committee, their perspectives have been extremely valuable to inform the programme theory development.

### Document characteristics

**Fig. 2:**
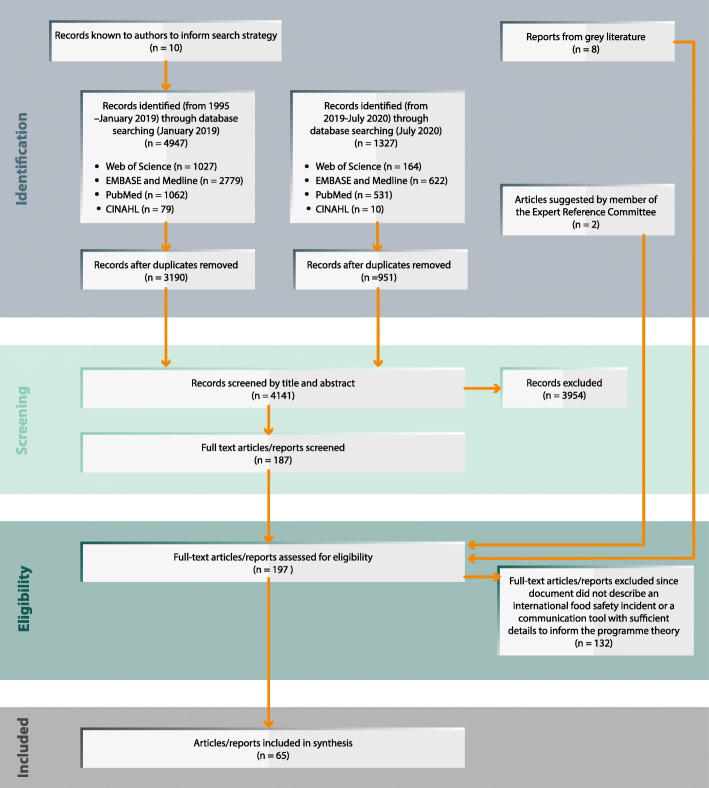
Flow diagram of the search strategy

Given the wider focus of what constitutes relevant evidence in a realist review, a total of 4141 articles were found across the databases after duplicates were excluded. Of these articles, 55 met the inclusion criteria. Additionally, eight relevant documents were found in the grey literature search, and two relevant documents were suggested by a member of the expert reference committee resulting in a total of 65 documents included in this review. For a flow diagram of the search strategy, see Fig. 2. The 65 documents retained fall under the broad categories of outbreak report [29], commentary [15], policy document [8], research article [6], review article [6] and meeting report [1].

### Communication tools to facilitate cross-border communication during international food safety events

**Fig. 3:**
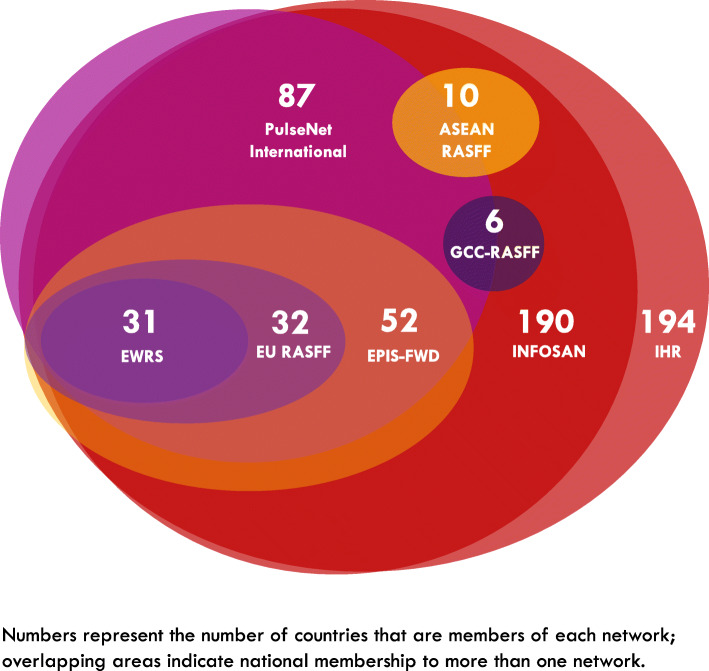
Networks/systems/tools currently in use to facilitate cross-border communication during international food safety events

A total of eight different tools to facilitate cross-border communication during international food safety events had been used in the reviewed documents and are summarised in Table 2. All of these tools utilise web-based platforms to facilitate information exchange among designated participants from government authorities. Figure 3 depicts the tools currently in use to facilitate cross-border communication during international food safety events and illustrates where overlaps between different networks exist from a national perspective. These overlaps do not take into consideration that different networks may include participants from the same country but different national agencies. For example, all 10 Member States from the Association of Southeast Asian Nations (ASEAN) have designated contact points as members of the International Food Safety Authorities Network (INFOSAN); however, some contact points for the ASEAN Rapid Alert System for Food and Feed (RASFF) are from national authorities that are not represented in INFOSAN.

**Table 2:** Communication tools to facilitate cross-border communication during international food safety events

Tool/System	Year Established	Who is using the tool?	Coordinating Authority	What is the purpose?	Key reference
European Union Rapid Alert System for Food and Feed (RASFF)	1979	EU Member State national food safety authorities, Commission, EFSA, ESA, Norway, Liechtenstein, Iceland and Switzerland	European Commission	Provide food and feed control authorities with an effective tool to exchange information about measures taken responding to serious risks detected in relation to food or feed	[31]
The International Molecular subtyping network for Foodborne Disease Surveillance (PulseNet International) Note: PulseNet International is a network of PulseNet national and regional networks	1996	National, regional and sub-regional laboratory networks of Africa, Asia Pacific, Canada, Europe, Latin America and the Caribbean, the Middle East, and the US in 86 countries	US Centers for Disease Control and Prevention (US CDC)	Implement standardised genotyping methods and share information in real-time within regional and national laboratory networks to support surveillance and outbreak response enabling the direct comparison of inter-laboratory data irrespective of geography	[32]
Early Warning and Response System (EWRS)	1998	Public health authorities from 30 countries including 27 EU Member States and three countries of the European Economic Area (EEA), Iceland, Norway and Liechtenstein.	European Commission	A rapid alert system to communicate serious cross border threats to health according to the Decision 1082/2013/EC between EU/EEA Member States, the European Commission, other EU agencies and WHO; EWRS is the primary risk management tool for international or unexpected events in the EU/EEA	[33]
FAO/WHO International Food Safety Authorities Network (INFOSAN)	2004	National authorities from 190 FAO/WHO Member States	FAO/WHO	Halt the international spread of contaminated food, prevent foodborne disease outbreaks, and strengthen food safety systems globally to reduce the burden of foodborne diseases	[15]
Association of Southeast Asian Nations Rapid Alert System for Food and Feed (ASEAN RASFF)	2007	National regulatory authorities from 10 countries in south-east Asia including Brunei, Cambodia, Indonesia, Laos, Malaysia, Myanmar, the Philippines, Singapore, Thailand, and Vietnam	National Bureau of Agricultural Commodity and Food Standards (ACFS), Ministry of Agriculture and Cooperatives, Thailand	Promptly exchange information among competent authorities when food or feed safety events occur	[34]
International Health Regulations Network of National IHR Focal Points (IHR)	2007	National Health authorities from 194 WHO Member States	World Health Organization (WHO)	Prevent, protect against, control and provide a public health response to the international spread of disease in ways that are commensurate with and restricted to public health risks, and which avoid unnecessary interference with international traffic and trade (considers all hazards, not only foodborne hazards)	[35]
Epidemic Intelligence Information System for food- and waterborne diseases and zoonoses (EPIS-FWD)	2010	Public health authorities from 51 countries including 27 EU Member States, three countries of the European Economic Area (EEA), Iceland, Norway and Liechtenstein plus 21 other non-EU countries	European Centre for Disease Prevention and Control (ECDC)	Detect multi-country food- and waterborne disease outbreaks and assessment of the public health risk. Note: The European surveillance network for *Salmonella* infections (Salm-Net) was established in 1994 in the UK and was later expanded to include other intestinal pathogens and renamed Enter-Net [36]; Enter-Net was transferred to ECDC in 2007 from the Health Protection Agency in the UK (now called Public Health England), and the scope of the network was broadened; it was renamed food- and waterborne diseases and zoonoses network (FWD-Net). The FWD-Net members exchange information on unusual increases of food- or waterborne diseases via the EPIS-FWD online platform, which is an informal system based on voluntary use. When the hypothesis for a specific food is strong, access to the EPIS-FWD will be granted to EFSA, the European Commission food safety unit (including RASFF) and the European Union Reference Laboratories. In some countries, veterinary users have been trained and can be granted access to selected events. The system is linked to the PulseNet International through US CDC and the Public Health Agency of Canada. In case of involvement of third countries, access is granted to WHO.	[33]
Gulf Cooperation Council Rapid Alert System for Food and Feed (GCC-RASFF)	2015	National Authorities from six GCC countries including Saudi Arabia, Kuwait, the United Arab Emirates, Qatar, Bahrain, and Oman	Secretariat General of the GCC; members of the GCC-RASFF use an electronic platform operated by the Saudi Food and Drug Authority, Kingdom of Saudi Arabia (SFDA)	Provide means for rapid exchange of information between GCC states on food alerts and food scares, flagging implicated food products to allow prompt regulatory actions. Note: the GCC-RASFF technical regulation has been submitted to the Arab Food Safety Initiative for Trade Facilitation (SAFE) for consideration by 17 Arab countries for adaptation to an ‘Arab RASFF’ to be coordinated by the League of Arab States (LAS).	[37]

### Contextual factors that trigger mechanisms to influence the outcomes observed in relation to the use of different communication tools

#### Country has interests in importing or exporting food commodities

Countries everywhere rely on internationally traded foods to meet consumer demands and feed growing populations [38]. Net exporting countries have an economic interest in ensuring the food they produce is safe and net importing countries have an interest in ensuring that food brought into the country is not contributing to the ill health of their population. As such, a country’s level of food import and export may influence the degree to which national authorities see a need to utilise specific tools to communicate about unsafe food in an international context. Savelli et al. (2019) have reported a positive correlation between the value of both food product imports and exports and involvement in food safety events communicated through INFOSAN between 2011 and 2017 for example [15]. In the Middle East, the Gulf Cooperation Council (GCC) countries import approximately 33 million tons of foods annually, estimated as 90% of their needs for food. This heavy reliance on imports has been described as one important factor driving the development and use of the GCC-RASFF by these countries [37]. The European Union (EU) is the largest global exporter of agri-food products, with a value of 151.2 billion Euros in 2019 (an increase of 10% from 2018). The EU also has a growing import agri-food product market, up 2.5% to 119.3 billion Euros in 2019 compared to 2018 [39]. With so many agri-food products being traded, and because of the European single market, products easily move between countries within the EU, necessitating the use of a system like RASFF to communicate on urgent international food safety issues [31]. In other regions, countries that are net importers of food may use INFOSAN as a practical platform to support their efforts in ensuring a safe domestic food supply [38].

#### Country has the technical infrastructure to detect food safety event (including foodborne disease outbreaks or food contamination) and conduct investigations

The prevention, control and mitigation of food safety risks rely on systems to be in place (e.g. an integrated surveillance system for foodborne diseases or food contamination monitoring program) to rapidly detect signals that suggest a potential risk to health as well as communication of the appropriate information to risk managers [40]. When such food safety risks are international, the currency exchanged between stakeholders includes data and information that stem from the epidemiologic, laboratory and traceability activities that are undertaken to assemble evidence during investigations. When one or more categories of evidence are deficient, then confidence in the appropriateness of subsequently applied risk management measures may be diminished. Furthermore, if the capacity to collect such evidence is limited or non-existent in one of these areas, then the ability to participate in international discussions related to such issues will also be similarly diminished. Unfortunately, foodborne diseases are often chronically under-reported in many parts of the world and subsequently under-recognised and deprioritised in terms of the allocation of resources or strategies for their prevention, control and reporting [41]. When foodborne disease outbreaks do arise, their successful management requires a well-structured food control system along with good communication, technical capacity, and access to information across all relevant sectors [7]. As articulated by Hodges and Kimball (2005) [42], international communication networks can serve as invaluable tools for collaboration and support, but their ultimate effectiveness is linked to individual nation’s capacities for surveillance and diagnostics related to food safety and foodborne disease. Functional participation in international networks engaged in food safety information exchange is supported when national food control systems are strengthened more generally [3].

#### Country is governed in accordance with regional and/or international laws and regulations relating to food control and global health security

Under the International Health Regulations (IHR (2005)) which came into force in 2007, all 194 Member States of the WHO have committed to a minimum set of national core capacities to protect public health and contribute to global health security [35]. INFOSAN is recognised as a fundamental tool to assist countries in developing the core capacities required for food safety emergency preparedness and response under the IHR [43]. While participation in INFOSAN is voluntary (190 Member States participating), the IHR (2005) provide a legally binding framework for the coordination of events that may constitute a public health emergency of international concern and for improving the capacities of countries to manage public health risks, including those posed by unsafe food [35]. In 2016, in recognition of the growth and development of INFOSAN, the Codex Alimentarius Commission (CAC) revised the “Principles and Guidelines for the Exchange of Information in Food Safety Emergency Situations (CAC/GL 19-1995),” by making appropriate references to INFOSAN [24]. This revision, endorsed by all CAC members (188 Member States), has further solidified the global mandate of INFOSAN and the critical and internationally recognised role that INFOSAN should play in the rapid exchange of information between countries during food safety emergencies. Other countries, in addition to being state parties to IHR, and members of INFOSAN and CAC, are also subject to regional legislation, as is the case of EU Member States. In the EU, food business operators (including importers) are legally required to ensure that upon investigation, traceability can be assured at all stages. This requirement is outlined in EC Regulation 178/2002, which lays down the general principles and requirements of food law in the EU and outlines the legal basis of RASFF in Article 50 [44]. In this way, national authorities from different countries in different regions are bound by separate agreements and legal frameworks that can mandate or encourage them to utilise different international communication tools.

### Mechanisms that influence the outcomes observed in relation to the use of different communication tools

#### Trust in fellow network members to maintain confidentiality where required and to apply measures that are proportionate to risk

Ensuring trust among stakeholders is an important mechanism to facilitate international information exchange and collaboration, between sectors and across borders to ensure global health security [35]. In their 2007 review of multi-national foodborne outbreak response, Ammon and Tauxe determined that utilising tools to communicate on multi-national foodborne disease outbreaks can largely depend on the trust among foodborne disease experts in different countries and their willingness to share information [45]. More recently, during an international meeting of members of INFOSAN in 2019, trust among members was reported as an essential factor that supports information exchange between countries on matters of food safety. It was also noted that while creating a trustworthy collaborative environment takes time, it is critical to building a strong community of practice among members [46].

#### Experience with different tools leading to institutionalisation of processes and procedures

Many of the articles included in this review that describe an international food safety event refer to the utility of RASFF, as a well-established system, in use since 1979 (longer than any of the other systems described in Table 2) [47, 48]. However, even with this long history, upon analysing notifications to the RASFF system from 1980 to 2017, Piglowski has noted that the activity of individual members of the RASFF can depend on members’ experience with the system [49]. When members have more experience with a particular communication tool and become more familiar with the requirements for engagement, then the processes and procedures can become institutionalised within their authorities, and the use of these tools becomes more regular. Following an international outbreak of *Salmonella* Enteritidis infections affecting three European countries in 2015, investigators reported that information exchange and access to systems such as EPIS-FWD are essential for collaboration during international investigations. However, they explained that clear guidance should be provided on how, when and what data to upload to such a system, emphasising that a lack of experience with protocols and procedures can limit collaboration [50]. In some cases, collaborating on an international investigation into a multi-country food safety event can provide experience to authorities in countries that are perhaps less used to doing so. With such experience comes the mindset that such collaboration is essential and needs to be maintained and reinforced [51]. Following an outbreak of Listeriosis in Switzerland in 2011 linked to imported cooked ham, investigators cited the critical role of RASFF to enable the rapid exchange of information between European countries. However, it was noted that even closer cross-border information sharing (e.g. sharing information on bacterial isolates) would have been helpful, but observed that when such forms of international cooperation were not institutionalised, communication was dependent on the goodwill of participating authorities [47].

#### Support from high-level government officials for participation in international communication activities (with clear roles and responsibilities agreed)

Gaining support from high-level government officials for the participation in international communication activities, with clear roles and responsibilities agreed (including agreement on the type of information to be shared), has been identified as a key element required to improve international cooperation and collaboration using established systems such as INFOSAN [35]. Food safety investigations rely on the willingness of multiple agencies involved within various countries to share information and to collaborate [52] and without senior or management level support, technical staff may not feel empowered to share information. The investigation into a large and prolonged outbreak of hepatitis A virus (HAV) infections in several European countries in 2013 and 2014 demonstrated the importance of having strong management support and coordination capability at the national level, noting that administrative hurdles and communication problems can lead to delays in notification of events [53]. Following an international outbreak of *Salmonella* Enteritidis infections affecting three European countries in 2015, investigators emphasised the importance of having clear roles and responsibilities assigned and supported by senior officials during international outbreak investigations, especially because of the substantial coordination required [50]. In the past, high-level political buy-in and prioritisation of food safety issues have often gained momentum in the face of large-scale food safety crises, for example in China following the melamine event in 2008 [7] and the United Kingdom following the announcement of the link between bovine spongiform encephalopathy (BSE) and the human form, variant Creutzfeldt-Jakob Disease (vCJD) in 1996 [54]. In both cases, the international implications and public concern triggered high-level government action and development of new initiatives to improve food safety. However, national authorities need not wait for a national food safety crisis before seeking high-level support for their efforts to prioritise food safety collaboration at an international level. If not already in place, support from high-level government officials and political buy-in for participation in international communication activities is often obtained during the development or exercising of a national food safety emergency response plan [55]. Such a plan should clearly outline the roles and responsibilities of different agencies in a food safety emergency response and high-level support from each of those agencies can help to ensure efficient cross-sectoral collaboration and communication across borders if and when required [55].

#### Awareness of the needs and requirements to collaborate and communicate across borders to ensure food safety

Information sharing, including disease reporting, at an international level under the International Health Regulations (2005) requires a host of stakeholders from multiple sectors to be fully trained and aware of their roles and responsibilities [56]. Awareness can come from sharing of expertise and experiences among stakeholders involved in international food safety events and is described as being an important and supportive factor during the international investigation into a large outbreak of HAV in Italy in 2013 and 2014 linked to imported frozen mixed berries [53]. Awareness is often improved as a result of the receipt of alerts that are disseminated through systems like INFOSAN, RASFF, or EPIS-FWD as was the case during an international outbreak of *Salmonella* Heidelberg infections associated with a meal during an international flight in 2011. Without awareness of the multi-country dimensions of the outbreak, disparate authorities may have assumed that identified cases were sporadic and not part of a larger, multinational outbreak. In this case, without such awareness, officials in Tanzania (the origin of the flight) would not have been provided with multiple lines of evidence which helped facilitate their domestic investigation [51]. In 2001, an outbreak of *Salmonella* Stanley infections occurred in Australia, Canada and the UK, resulting in 109 cases of illness, that were linked to the consumption of internationally distributed peanuts from a fourth country in Asia [57]. Control of this outbreak relied on rapid communication of findings between investigators, including isolate characteristics as well as epidemiologic and traceback information. Investigators suggested that as a result of this outbreak investigation and international collaboration, they had a greater awareness of the benefits of sharing information through collaborative networks during subsequent investigations [57]. Multi-national food safety events emphasise the needs and requirements to collaborate and communicate across borders but also highlight the fact that food safety is sometimes a hidden and often overlooked problem except in the face of a crisis. Sustained efforts to raise awareness about the importance of food safety as a public health problem with a focus on prevention are required at all levels of society and government alike [58].

#### Understanding that open communication during international food safety events contributes to global public health

Following an investigation in 2007 of an outbreak of shigellosis in Denmark and Australia linked to imported baby corn from Thailand, investigators reflected on the crucial role of several networks to facilitate worldwide communication on various aspects of the investigation including EPIS-EWRS, RASFF, PulseNet International and INFOSAN. Investigators underscored the importance of involved international stakeholders, having an understanding that open communication between countries worldwide can lead to timely responses, improved public health and prevention of similar outbreaks in the future [59]. In their discussion about food safety issues in the Maghreb Area, Chammem et al. [60] explain that understanding the importance of open communication between the different actors across the food supply chain is paramount for the timely management of risks and control of hazards, especially during food safety emergencies when INFOSAN can be used for the effective sharing of information and promotion of collaboration at national and international levels.

#### Sense of community among fellow network members

In different parts of the world, various regional communication tools have developed to link together national authorities from countries that share a common language (e.g. GCC-RASFF), geographic region (e.g. ASEAN RASFF) or other factors that contribute to a sense of community like a common legal system and similar levels of development, societal and cultural norms and industrial structure (e.g. EU RASFF). Reflecting on INFOSAN, a member of the network from Thailand described how participating in INFOSAN reduced the distance between each participating country and creates one united community for food safety that enables the sharing of information for action on food safety risk management in a timely manner [22]. As explained by Savelli et al., INFOSAN members share common responsibilities and undertake activities with shared goals in mind which creates a sense of community. Members of INFOSAN participate in network activities, to exchange information with each other across borders, to improve food safety and deepen their knowledge and expertise in the area by learning from one another and regularly interacting [15].

#### Standardisation

During international foodborne disease outbreaks, there is an inherent reliance on the comparability of data in order to determine if disparate cases of illness are related. In this way, standardisation is essential in terms of molecular methods for comparing foodborne bacterial strains, for example [52]. During the investigation into cases of *Salmonella* Goldcoast in Italy and Hungary in 2009 and 2010, implementing the use of standardised protocols for *Salmonella* strain typing between human and veterinary laboratories was critical in order to generate hypotheses about a possible zoonotic connection of the outbreak cases in both countries to the pork production chain [61]. During an outbreak investigation of *Salmonella* Enteritidis infections in several European countries in 2014 linked to eggs, investigators utilised RASFF to exchange information between countries and combined whole genome sequencing (WGS) data with information on food distribution networks to facilitate a more detailed exploration of possible sources of infections and inform risk management measures. Investigators emphasised the need for further work be undertaken to develop and standardise the methods used to compare phylogenetic and food supply network information, to enable the use of these techniques in future international outbreaks to help identify sources and guide the implementation of control measures to prevent further illness [62]. During the investigation, an important factor that enabled data to be readily exchanged and analysed between four institutions in different countries was the digital nature of the data [62]. When different typing methods are used between countries or sectors (e.g. WGS-based methods, Multiple Locus Variable-number Tandem Repeat (MLVA) analysis, Pulsed-field Gel Electrophoresis (PFGE) analysis, or no subtyping), it introduces challenges for investigators that hinder efficient communication related to the identity of isolates and limits the ability to link international cases together [63]. Two decades ago, the technology may have been different, and there was a heavier reliance on PFGE rather than WGS, but the idea of using standard methods for PFGE and setting up compatible networks on a global scale was already being discussed, particularly as PulseNet USA had demonstrated its utility. Following an outbreak of *Salmonella* Typhimurium infections in several European countries in 2000 linked to shredded lettuce, investigators highlighted the importance of standardised protocols for molecular typing, and they emphasised the need for compatible networks for the exchange of electronic, molecular data in real-time [64]. More recently, standardisation concerning protocols, validation studies, quality control programs, database development, and training materials has been highlighted as a critical element for PulseNet International in order to facilitate the sharing of data and information internationally and the implementation of WGS for global foodborne disease surveillance [32]. Additionally, the ECDC has facilitated standardisation of MLVA techniques for *Salmonella* Enteritidis and *Salmonella* Typhimurium, which are the most commonly reported *Salmonella* infections in EU/EEA [65]. The standard methods facilitated the detection of cross-border spread of *Salmonella* infections due to contaminated eggs from Poland, which was communicated about through RASFF, EPIS-FWD and PulseNet International [63].

#### Intersectoral collaboration

Utilising data from epidemiological studies, laboratory investigations of food and clinical samples, as well as data and information from traceback or trace-forward activities, is an integral part of investigating food safety events and supports the use of international communication tools, as demonstrated in multiple outbreak reports [48, 52, 53, 66]. For example, upon investigating three simultaneous outbreaks of HAV infections in Europe in 2013, Gossner and Severi [48] emphasised the necessity for extensive international collaboration between countries and intersectoral collaboration between public health and food sectors in order to identify possible vehicles of infection and implement timely control measures. Systems, including RASFF and EPIS-FWD, were utilised during these outbreaks to exchange information and helped to distinguish cases into three distinct outbreaks as well as to strengthen various hypotheses by pooling data and information from multiple countries. Similarly, following a multi-country outbreak of *Salmonella* Stanley infections in Europe linked to turkey meat, investigators described how intersectoral collaboration across public health, veterinary and food sectors enabled timely implementation of control measures and information sharing through EPIS-FWD and INFOSAN. Specifically, it was mentioned that involving multiple sectors in the investigation enabled the collection of robust evidence pointing towards the turkey production chain and confirmed the emergence of a new microbial clone within Europe [66]. Such intersectoral activities involve the integrated effort of multiple disciplines working to attain optimal health for people, animals, and the environment, also known as One Health [35]. A primary challenge for effectively responding to outbreaks of foodborne zoonoses is ensuring collaboration and coordinated planning across sectors while harnessing the available technologies. Taking this kind of One Health approach calls for collaboration across disciplines, sectors, organisations, and national borders in support of increasingly complex health challenges, including international food safety events [67]. However, processes involved in the planning and implementation of intersectoral actions are complex, and each country needs to develop or review its own strategy for intersectoral action which can support the use of international communication tools during food safety event response [68].

### Outcomes observed in relation to the use of different communication tools by competent authorities to relay information about international aspects of food safety events abroad

#### Efficient exchange of information among international stakeholders

Nearly twenty-five years ago, collaboration on international analytic studies during multi-country foodborne disease outbreaks was in its infancy [69] often occurring through informal networks [36], but was nonetheless recognised as necessary for detecting related clusters of foodborne illness and identifying widely distributed contaminated foods [70, 71]. In 1995, a then newly-established, international *Salmonella* surveillance network assisted investigators in solving an outbreak of *Salmonella* Agona with infections in the UK, USA, and Israel [72]. During the response, investigators recognised the crucial role of international communication networks in facilitating efficient information exchange within Europe and beyond, which in this case led to the identification of the source of the outbreak and the swift implementation of risk management measures in multiple countries [72, 73]. Similar outcomes were reported following an outbreak of *Salmonella* Anatum infections in France and the UK in 1997 during which rapid communication was facilitated by the same international *Salmonella* surveillance network [74]. Since outbreak reports such as these first started to demonstrate the value of international collaboration, responses to international food safety events have continued to highlight the usefulness of establishing and maintaining information-sharing networks globally that enables the rapid exchange of information between food regulatory agencies worldwide [52, 63, 75–78]. For example, in 2002, an outbreak of norovirus infections in Italy and France was linked to oyster consumption, resulting in 327 cases between the two countries. Investigators credited the existence of an international foodborne virus laboratory network in Europe for facilitating information sharing rapidly and efficiently in order to track the international spread of the virus and lend assistance for the interpretation of results during the international investigation [75]. Just over a year later, an outbreak of norovirus infections in Australia in 2003 and 2004 was linked to imported oyster meat from Japan [76]. Investigators concluded that information sharing across borders provides countries with the intelligence required to develop effective control strategies. They also noted that INFOSAN, a tool that had just launched at that time, would be useful in disseminating such information on a global scale [76]. More recently, it has been noted that utilising systems like RASFF and INFOSAN creates a network of partnerships that enables the efficient exchange of information during international food safety events [77]. Jansen et al. (2016) have reported that because of the efficiency of RASFF, serious harm to consumers in Europe has been avoided, mitigating the negative health impact of food safety crises [78]. For example, during an international outbreak of *Salmonella* infections linked to eggs from Poland, the utilisation of RASFF, EPIS-FWD and PulseNet enabled the rapid exchange of information internationally between public health authorities [63]. During the investigation of a foodborne outbreak of *Shigella sonnei* infections in Ireland and Northern Ireland in 2016, cross-border information sharing using EPIS-FWD facilitated the efficient identification of the outbreak, the early generation of a hypothesis and the rapid implementation of control measures [52].

#### Timely detection, notification, investigation and response to food safety events (including the implementation of risk management measures)

The utilisation of international networks including EWRS, PulseNet RASFF and INFOSAN helps to facilitate timely international communication to identify when a contaminated food enters international trade, enabling the implementation of risk management measures by competent authorities, to prevent foodborne disease [59]. For example, using EPIS-FWD allowed the early detection of the multinational nature of three distinct outbreaks of HAV infections in Europe in 2013 [48]. Referring to the same outbreak investigations, officials from Italy also noted the utility of EPIS-FWD and the crucial role it played to facilitate information exchange at a regional level to facilitate outbreak detection and investigation [53]. Following a multi-country outbreak of *Salmonella* Bovismorbificans infections in Switzerland and Germany in 2014, investigators credited the cross-country collaboration for timely identification of the source as well as prevention of an expanded outbreak, thereby protecting public health [79]. Following an outbreak of *Salmonella* Typhimurium infections in Denmark, Norway and Sweden in 2008, investigators concluded that utilising international communication tools (in this case, EPIS-FWD), supported by strong intersectoral collaboration and harmonised molecular typing tools, allowed for the practical identification and management of the outbreak in the neighbouring countries [80]. Utilising PulseNet, RASFF, EWRS and INFOSAN allowed for information related to food surveillance, molecular microbiology and epidemiology to be gathered quickly and disseminated effectively during an international outbreak investigation in 2007 involving cases of *Salmonella* Senftenberg infections in several European countries as well as the USA linked to basil from Israel [81]. During an outbreak of *Salmonella* Typhimurium DT104 infections in Denmark in 2005 linked to imported carpaccio from Italy, investigators utilised RASFF to communicate internationally about the details of contaminated batches of carpaccio, alerting other importing countries of the problem and implement timely risk management measures. In this case, investigators also emphasised how this outbreak illustrates the increasing importance of international cooperation during such events [82]. Widespread outbreaks caused by low-level contamination of foodborne pathogens can be challenging to identify. However, when information is readily exchanged using international tools such as PulseNet, EWRS and RASFF, such outbreaks are more quickly detected and investigated as demonstrated during a widespread outbreak of *Salmonella* Thompson infections in Norway, Sweden and the United Kingdom linked to rucola lettuce from Italy [83].

#### Robust understanding of international dimensions of a given food safety event and documentation of lessons learned

Utilising international communication tools to share information during multi-national food safety events enables detailed documentation by international agencies to fully understand the scope of a given food safety event. Detailed documentation can help with the recording of lessons learned and the sharing of best practices to a broad audience to prevent similar events in the future or to make the handing of an acute event more efficient. For example, a prolonged international outbreak of *Salmonella* Enteritidis infections affected 18 European countries between 2015 and 2018 and was eventually linked to eggs from Poland [63]. The successful identification of the source of this outbreak, as well as the association of cases from multiple countries to the same source, was only made possible through cross-border sharing of data and information in real-time through various systems including EPIS-FWD, RASFF, and PulseNet. Without international information exchange facilitated through these platforms, the full scope of the outbreak would not have been known [63].. Upon review of foodborne outbreaks in the USA from 2010 to 2014, many of which were linked to imported foods, Crowe et al. [84], have stressed the importance of collaboration between government and industry, specifically the utility of sharing lessons learned as a way to improve food safety practices and regulations and prevent future outbreaks. In 2011, a group of travellers returning to Ireland from Tanzania became ill with *Salmonella* Heidelberg infections. The authorities, investigating the Irish cases alone, could not definitively pinpoint the location of the outbreak or the source. Only through international collaboration, and by including information on cases from other countries in their study, were authorities able to pinpoint an in-flight meal and identify two items in particular as the likely source of infections. During the investigation, information was exchanged internationally using EPIS-FWD, EWRS and PulseNet and investigators emphasised the benefits of real-time international collaboration and the utility of these communication networks. Utilising such tools enabled the sharing of standard questionnaires, results from molecular profiling, hypotheses and other information that made the investigation more efficient and effective [51]. In another example, a multi-country outbreak of *Salmonella* Stanley infections in Europe occurred over several years from 2011 to 2013, highlighting the challenges in detecting and investigating food safety events involving a contamination event early in the animal production chain resulting in multiple vehicles of infection across multiple countries. However, by sharing data, information, investigation tools (e.g. standardised questionnaire) through systems including EPIS-FWD and INFOSAN, investigators were able to identify the source as turkey meat, most likely contaminated early in the production chain [66]. In 2008, the actions of nearly 70 countries were communicated through INFOSAN during the international response to the global distribution of milk and milk-containing products that had been deliberately contaminated with melamine in China. The rapid worldwide distribution of affected products affirmed the need for a system like INFOSAN to coordinate communication and link together food safety authorities to promote the rapid exchange of information. The international response to this event exemplified how sharing best practices can save lives and help to control an outbreak. Utilising INFOSAN during this event allowed food safety authorities around the world to have access to the latest available scientific knowledge as new information became available [7].

#### Reduction of food safety risks

International collaboration can lead to the reduction of food safety risks in the short term by identifying unsafe products to be recalled from the market, as mentioned in previous examples already discussed. However, it can also result in longer-term changes to policies and practices that reduce food safety risks. For example, in 2009 and 2010, a large outbreak of hepatitis A virus infections was reported in Australia and linked to the consumption of imported semi-dried tomatoes. Notification of the outbreak in Australia through INFOSAN enabled the identification of related hepatitis A clusters in the Netherlands and France, also linked to imported semi-dried tomatoes [85]. International cooperation through INFOSAN supported national investigations during this multi-country outbreak [85] and demonstrated the critical interface with European networks, including RASFF and EWRS through which information was also shared. This outbreak represents the first documented outbreak of HAV infections linked to semi-dried tomatoes and demonstrates the value in utilising networks like INFOSAN to share surveillance data and alerts between sectors and countries [86]. As a result of the global, coordinated action between countries, international attention was drawn to these events which prompted industry forums to improve manufacturers’ knowledge of the risks associated with products like semi-dried tomatoes and related mitigation strategies to reduce such risks in the long-term and prevent future outbreaks [85].

#### Prevention of foodborne disease around the world

Following the investigation into international food safety events, multiple reports have concluded that utilising different tools to facilitate cross-border communication has prevented foodborne illnesses and protected public health [79, 87–89]. For example, in 2007, 50 cases of *E. coli* O157 H- infections were reported in the Netherlands and Iceland and linked to the consumption of shredded, pre-packed lettuce from the Netherlands. The outbreak was first reported to other European countries by Iceland through ECDC’s FWD network, and the Netherlands responded with a report of a similar outbreak. Investigators concluded that by combining efforts, compiling and analysing data from both countries increased their ability to detect the source at an early stage and strengthened their epidemiologic evidence. Investigators also emphasised that cross-border collaboration, in this case, enabled earlier implementation of risk management measures and led to a decrease in both morbidity and mortality [87]. In another example from 2010, investigators in France determined that a large outbreak of *Salmonella* Typhimurium 4,5,12:i:- infections affecting more than 500 people was caused by consumption of beef imported from another European country. Utilising RASFF, authorities in France were able to exchange information about this event between authorities in the country of origin who were able to quickly identify and withdraw the implicated beef, thus preventing further infections in other countries in receipt of the incriminated batch of beef [88]. Upon analysing notifications to the RASFF system from 1980 to 2017, Piglowski has concluded that RASFF significantly contributes to ensuring public health by preventing illnesses caused by microorganisms in food, especially on the European market [49]. In late 2012, an outbreak of salmonellosis linked to the consumption of tahini from Turkey was investigated in New Zealand. A few months later, cases of *Salmonella* infections were identified in the USA with strains indistinguishable from the from New Zealand cases, confirmed through information exchange using PulseNet [89]. A global alert was subsequently shared through INFOSAN and authorities in Turkey were able to determine that the implicated tahini products were also distributed to 13 additional countries. Information shared through INFOSAN enabled competent authorities in recipient countries to recall products and prevent further outbreaks and protecting public health. Without INFOSAN, the international scope of this event would not have been realised, and information required by national authorities to take risk management actions to protect public health would not have been disseminated [22]. Following this investigation, a former INFOSAN member from New Zealand described INFOSAN as a valuable platform that operates efficiently and reliably to enable flexible communication arrangements that can be tailored to the needs and risk of food safety concern [22].

### Realist programme theory

**Fig. 4:**
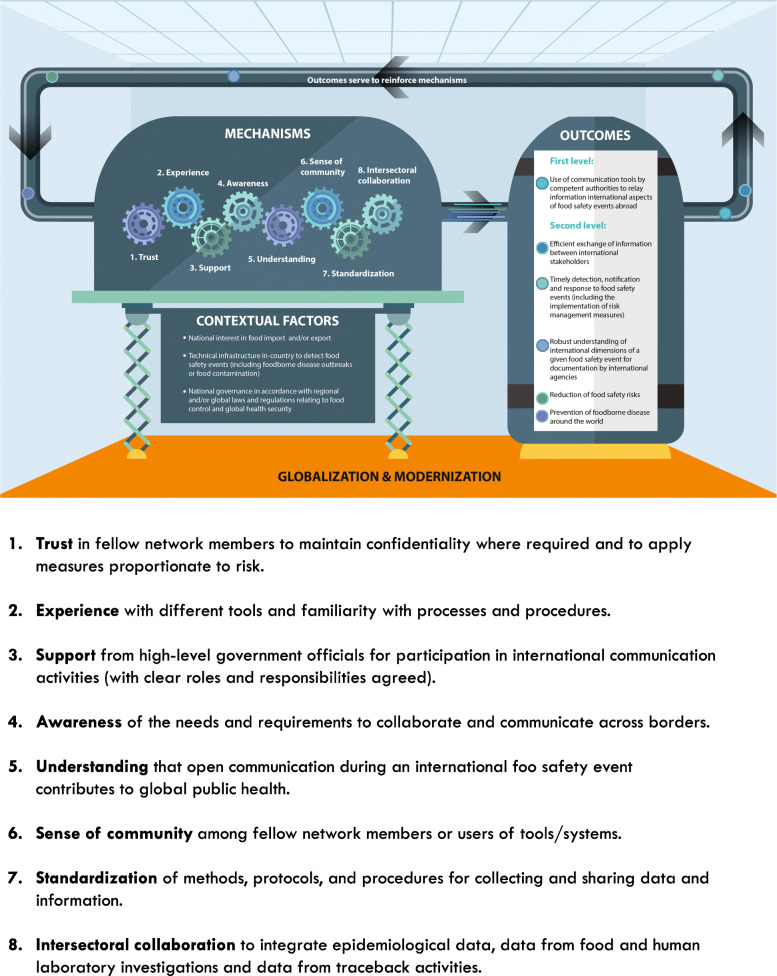
Realist programme theory to explain how different tools facilitate cross-border communication during international food safety events

The programme theory developed indicates that when a country has interests in importing or exporting food, has the technical infrastructure to detect food safety events, and is governed in accordance with regional and/or global laws and regulations relating to food control and global health security, then certain mechanisms lead to specific outcomes. These mechanisms, including trust, experience, support, awareness, understanding, a sense of community, standardisation and intersectoral collaboration will facilitate the first-level outcome of using communication tools to relay information abroad and a potential range of second-level outcomes, including the prevention of foodborne diseases, among others as described in Fig. 4. The programme theory developed includes a feedback loop whereby the act of using a communication tool can itself serve to reinforce each mechanism. For example, awareness of the tools facilitates their use, but using the tools also raises awareness about them. Likewise, trust among stakeholders can facilitate the use of communication tools, but using the tools can also build trust over time. A similar pattern for other mechanisms can be seen. The refined programme theory is underpinned by modernisation theory [27, 28] and globalisation theory [29], reminding us that efforts to modernise society occur at vastly different paces in different places and globalisation is not a linear process. Engaging with these theories provides us with an understanding that while the world is becoming ever more interconnected and interdependent, specific structures built to support development cannot be imposed in precisely the same way and at the same time in different countries since the country-specific context will influence the outcomes.

## Discussion

The findings of this review have illuminated a variety of communication tools that are currently in use around the world by various stakeholders in order to exchange information during international food safety events. However, there is a clear absence of published event reports which describe the use of some of the tools included in Table 2, namely the GCC RASFF and ASEAN RASFF. The absence of articles may be partially explained by the limit of this review to English publications and also by the fact that these tools are relatively new. However, there also remains the possibility that these networks have not matured to the point at which their utilisation has resulted in many successful collaborations during international food safety events. To better understand regional network proliferation and potential underutilisation, it is essential to consider the context. The EU RASFF system and other European tools work so well, in part, because there is a common legal system and similar levels of development, societal and cultural norms and industrial structure which is not the case for ASEAN countries, for example [34]. Interestingly, a study of the challenges for international data sharing among countries in the Greater Mekong subregion (Cambodia, Lao PDR, Myanmar, and Vietnam) found that differences in language, culture, surveillance systems and political engagement have all been reported as potential challenges to harmonising surveillance data between countries. Such differences can lead to variations in the quality of data reported, difficulty in data integration and comparison and interpretation [90]. A plan of action to make improvements to ASEAN RASFF was adopted at the ASEAN ministerial meeting on agriculture and forestry in 2018, which runs through 2023 [91]. Such efforts to improve this and other regional networks and tools would benefit from also considering the programme theory developed in this review and addressing issues related to the national context and the status of the various mechanisms identified to facilitate their use better.

As various regional networks and tools develop and are operationalised, it will be of paramount importance to link these tools to global systems such as INFOSAN in order to avoid parallel communication tracks or duplication of efforts by national authorities with limited resources. One way this can be avoided would be to ensure common contact points between regional and global networks and the formalisation of relationships through memorandums of understanding to ensure functional interfaces are in place. For example, the formal working instructions of European RASFF dictate when and how the INFOSAN Secretariat at WHO is informed of issues involving countries outside the EU [92]. This arrangement enables the INFOSAN Secretariat to follow up accordingly. Additionally, all RASFF members are also INFOSAN members. The functional interface between the two networks should be encouraged and replicated with other regional systems and tools that are in place or under development in other parts of the world to avoid resources from being allocated to disparate and disjointed tools which could hinder international food safety event coordination. Following multiple international outbreak investigations in Europe, investigators have emphasised that strong collaboration with existing international networks should be ensured [48]. The need for such functional links between INFOSAN and regional networks and systems including with ASEAN RASFF and GCC-RASFF were discussed at the second global meeting of INFOSAN members in 2019, and there was a clear recognition of the need to coordinate between systems and the critical role that INFOSAN can play in this regard [46]. Finally, it should be noted that the utility of any of these tools is dependent on the quality of the data and information supplied to them, and the speed at which users do this. In this regard, future systems and tools may benefit from the introduction of automation and validation to improve data quality, and increase the timeliness of the information exchanged to help identify potential international food safety events before they grow into large-scale crises.

### Strengths, limitations and future directions

Increasingly, globalisation of our food supply necessitates international communication and coordination among food safety and public health professionals to prevent, detect, and respond to foodborne disease outbreaks and instances of food contamination that affect more than one country. This review contributes to understanding how the various tools used to facilitate communication are working and in what contexts. The knowledge gained from this review has provided valuable insight into how different tools facilitate cross-border communication during international food safety events, why they are used, by whom, and for what purpose. One limitation of this review is that it was only conducted in English, introducing an element of language bias. Additionally, the formulation of the context-mechanism-outcome programme theory relies heavily on published literature and is subject to publication bias. Review findings are, therefore, context-specific and must be considered within the context of this research. While conducting this review and assessing the quality of research, it became clear that most published evidence in the area is anecdotal with subjective accounts from investigators involved in the use of various communication tools being the source of most of the evidence for utility. Also, much of the literature included in this review is very Euro-centric, even when the origin or distribution of implicated products in a given event is beyond EU borders. In the future, more effort to include the perspective of all countries involved in international food safety events, including those from which contaminated food originates, should be made when writing and publishing event reports. Including these perspectives would contribute to a gap in the literature and amplify the voices of those currently underrepresented but who would undoubtedly have valuable lessons to share with the global food safety community. With their global mandate, the FAO/WHO INFOSAN Secretariat is well-positioned to facilitate this kind of collaboration. Another role of the INFOSAN Secretariat would be to establish or strengthen functional links with current and future regional networks and tools to ensure complementarity between global and regional systems to prevent duplication or the creation of parallel communication tracks to the detriment of timely and coordinated global response efforts. Such work would align with the strategic directions for 2020–2025 outlined by the INFOSAN Secretariat [93] and should be informed by diverse perspectives from experts across all regions. Despite the gaps in the literature, this review draws strength from the engagement with an expert reference committee, whose members hail from a multitude of geographically diverse countries who provided oversight, guidance and rigour to the review process.

### Comparison with existing literature

Although slightly broader in scope, a review of early identification systems for emerging foodborne hazards conducted just over a decade ago concluded that little information had been published on the performance of operational food safety early warning or emerging risk systems [94]. Having searched for similar information, we have found the same deficiency in the literature more than ten years later with few empirical studies reporting on the impact of such systems. This deficiency may be partially explained by the difficulty in quantifying the impact of preventing severe food safety events without knowing what would happen if those systems or tools were not used. International food safety events are often responded to by taking a so-called, “One Health” approach since they require collaboration across disciplines, sectors, organisations, and national borders as described by Errecaborde et al. [67]. The global food safety community would benefit if investigators involved in future documentation and evaluation of such event responses utilised the 2019 framework developed by Errecaborde et al. [67] as part of their scoping review of factors that enable effective One Health collaborations. Using such a framework could result in more uniform and systematic documentation and perhaps allow for empirical evaluation of future studies of the utilisation of tools to facilitate cross-border communication during international food safety events in the literature.

## Conclusions

Responding to international food safety events is complex for several reasons, including because of the globalised nature of our food supply, the involvement of numerous international and national stakeholders, and the dependence on functioning national integrated surveillance systems and national food control systems more broadly. In this realist synthesis, a programme theory has been presented to explain how tools are utilised to facilitate cross-border communication during international food safety events which has important implications on global efforts to mitigate the significant burden of foodborne illness resulting from internationally distributed food. Overall, the results have shown that the various tools examined facilitate cross-border communication during food safety events by making functional connections between national regulatory authorities in different countries, supported by several specific mechanisms. The various tools are used because they facilitate, streamline or expedite national response efforts during food safety events, ensuring timely information exchange by those using them. The literature indicates that while nearly all countries around the world are members to one or more of the networks/systems/tools discussed, the European tools are very well used, while others in Asia and the Middle East are still maturing. Notably, all of the tools identified have overlapping national membership with INFOSAN, as per its global mandate to connect food safety authorities worldwide. The ultimate goal of all of the tools identified is to reduce foodborne risks and prevent foodborne diseases. The programme theory will be useful to policymakers and those coordinating the operation of communication tools currently in use, who may adapt components of the tools according to different contextual factors to promote, support and improve their use. Adaptation of the various tools to local contexts is fundamental in ensuring their utility. Also, the resulting programme theory should be considered as a useful framework to understand the study of INFOSAN which aims to fill a gap in the literature by providing evidence from a rigorous study of a specific tool that facilitates cross-border communication during international food safety events [95]. The programme theory would also be useful to inform future studies of other such networks and tools that have yet to be undertaken. All relevant national food safety authorities should be encouraged to actively make use of the various international tools available to them to openly exchange information and strengthen the global community of food safety practitioners. In doing so, national authorities will contribute to the strengthening of core capacities for food safety required under the IHR (2005), thereby improving global health security. The global burden of foodborne disease can be mitigated by improving international coordination and communication during international food safety events.

## Supplementary Information

**Additional file 1.** Details on the search strategy.

## Data Availability

Not applicable.

## Abbreviations

ASEAN: Association of South-East Asian Nations
CAC: Codex Alimentarius Commission
CMO: Context-Mechanism-Outcome
EEA: European Economic Area
EPIS-FWD: Epidemic Intelligence Information System for Food- and Waterborne Diseases and zoonoses
EU: European Union
EWRS: Early Warning and Response System
GCC: Gulf Cooperation Council
HAV: hepatitis A virus
IHR: International Health Regulations
INFOSAN: International Food Safety Authorities Network
MLVA: Multiple Locus Variable-number Tandem Repeat
PFGE: Pulsed-field Gel Electrophoresis
RAMESES: Realist and Meta-narrative Evidence Synthesis: Evolving Standards
RASFF: Rapid Alert System for Food and Feed
UK: United Kingdom of Great Britain and Northern Ireland
USA: United States of America
WGS: Whole Genome Sequencing
WHO: World Health Organization
