# Supplementary material for: The utilisation of tools to facilitate cross-border communication during international food safety events, 1995–2020: a realist synthesis
Source: Global Health. 2021 Jun 24;17:65. doi: 10.1186/s12992-021-00715-2 (PMC8222958; doi:10.1186/s12992-021-00715-2)

**Supplementary file 1 – Details on the search strategy**

**January 2019**

**Web of Science**

|  |  |  |  |  |  |
| --- | --- | --- | --- | --- | --- |
|  | | | | | |
|  |  |  |  |  |  |
|  | | | | | |
|  |  |  |  |  |  |
|  | | | | | |
|  |  |  |  |  |  |
| 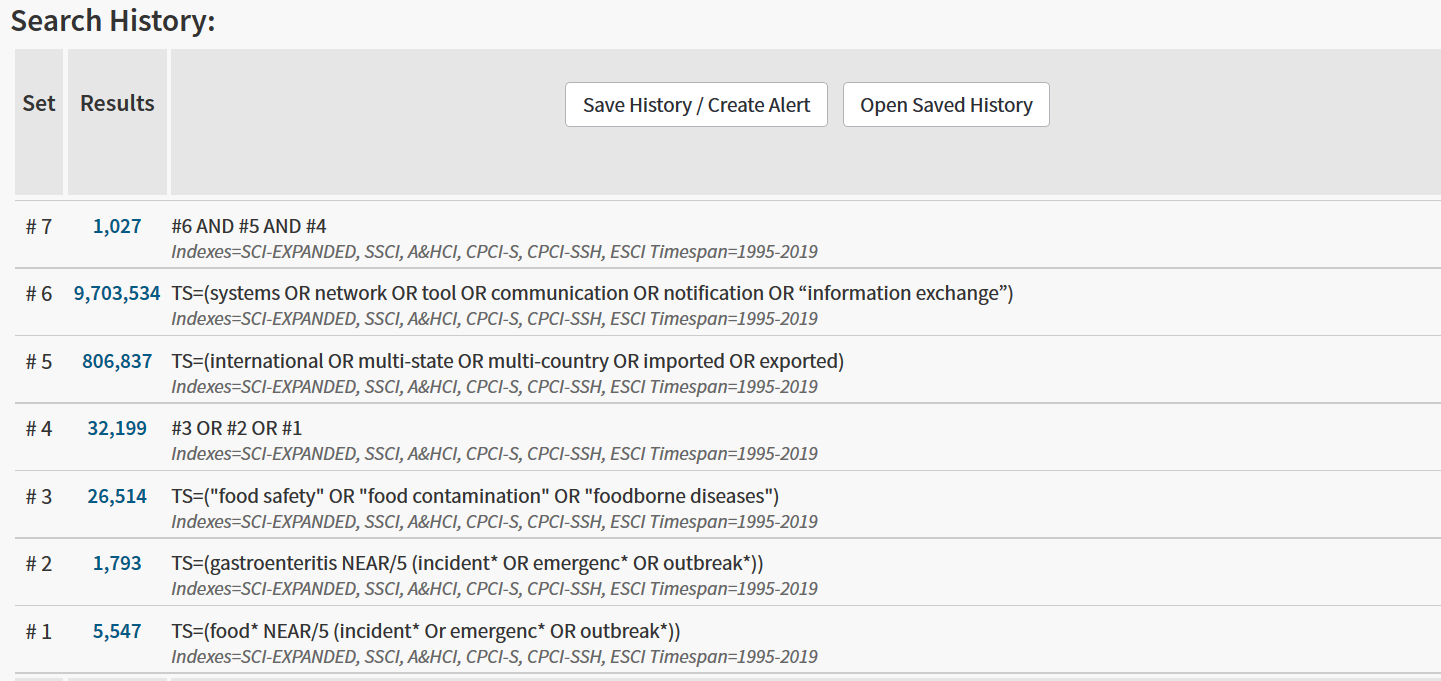 | | | | | |
|  |  |  |  |  |  |
|  | | | | | |
|  |  |  |  |  |  |
|  | | | | | |
|  |  |  |  |  |  |

**Embase and MEDLINE**


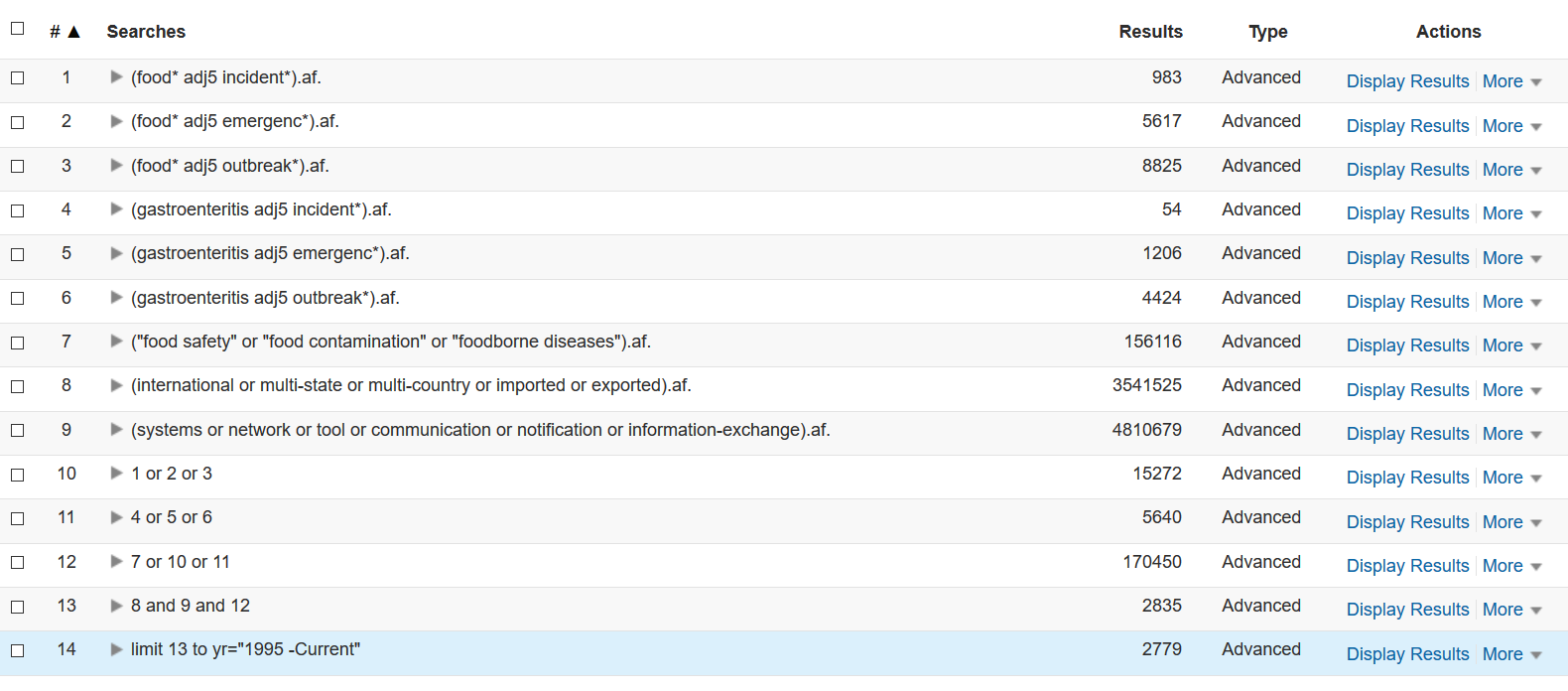


**PubMed**

**PubMed**


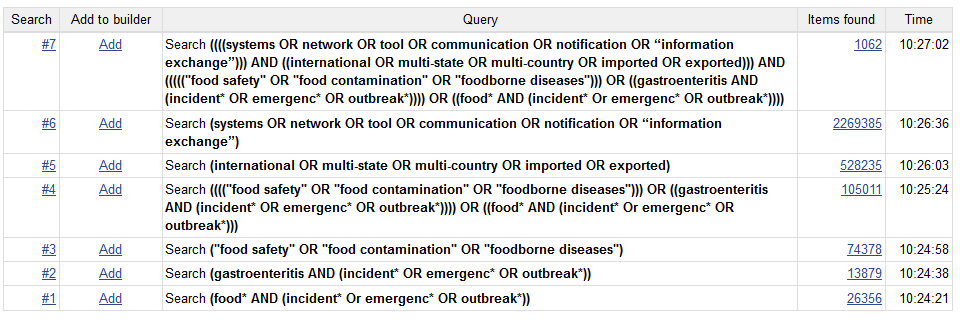


**CINAHL (with proximity operators; syntax: n5)**


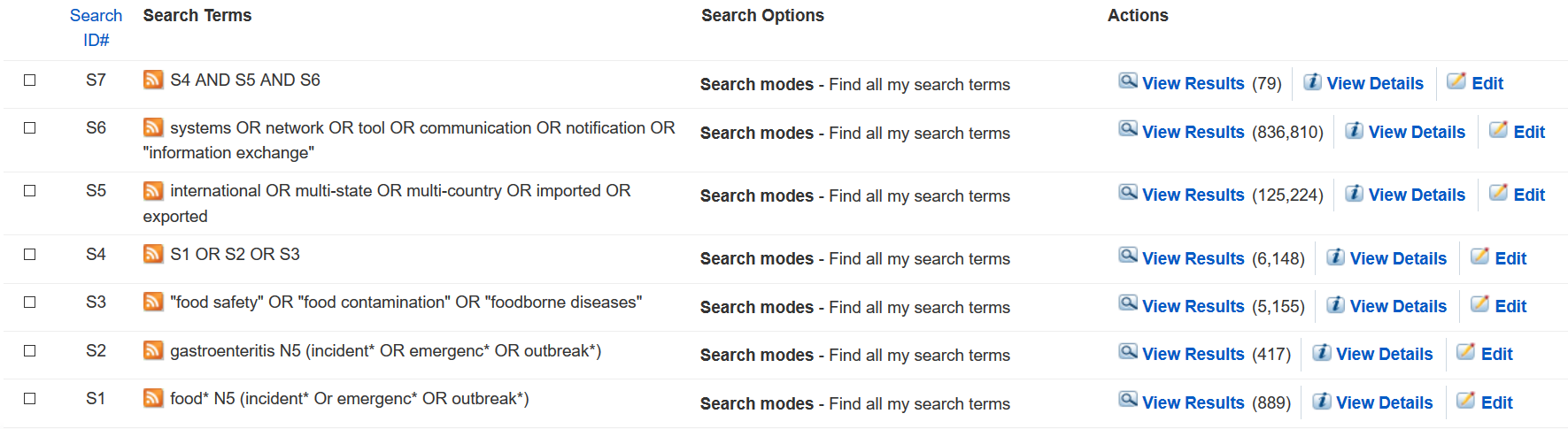


**Re-Run Searches 17 July 2020**

**Web of Science**


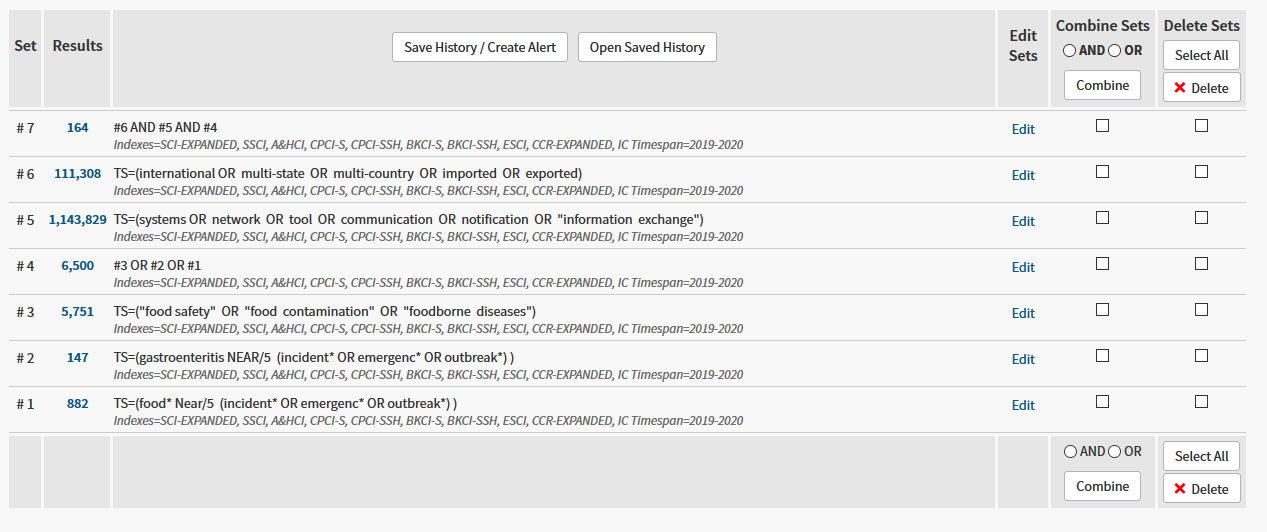


**Embase AND MEDLINE**


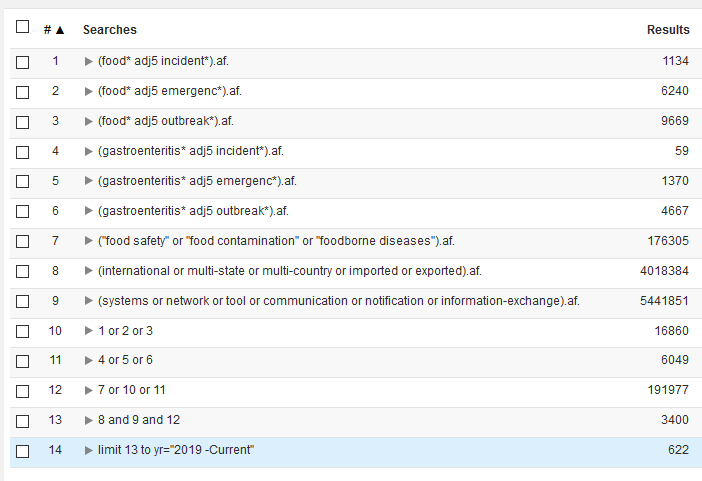


**Pubmed**


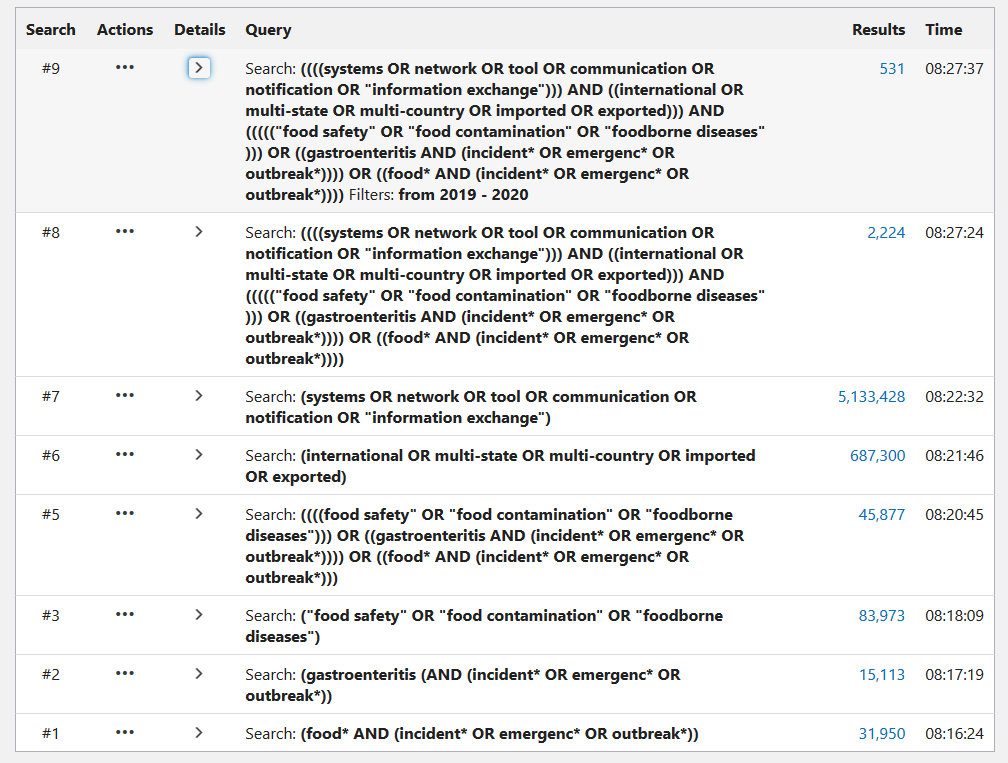


**CINAHL (with proximity operators; syntax: n5)**


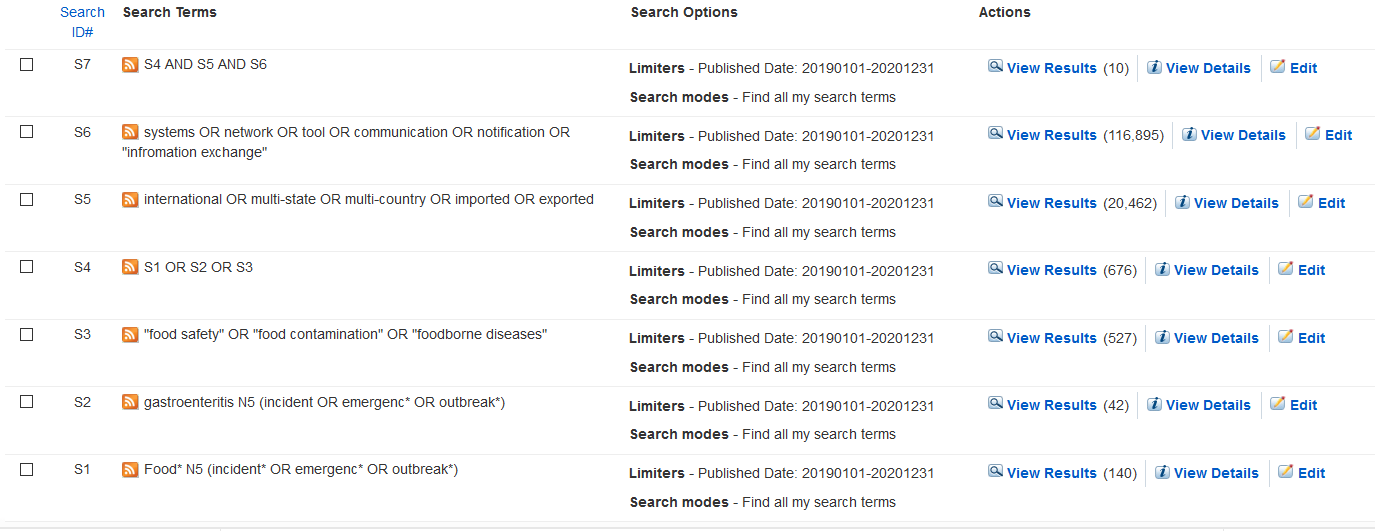

Supplement: Supplementary file 1 — Additional file 1. Details on the search strategy. [file 12992_2021_715_MOESM1_ESM.docx]
